# Autoimmunity as a Double Agent in Tumor Killing and Cancer Promotion

**DOI:** 10.3389/fimmu.2014.00116

**Published:** 2014-03-18

**Authors:** Kevin H. Toomer, Zhibin Chen

**Affiliations:** ^1^Department of Microbiology and Immunology, University of Miami Miller School of Medicine, Miami, FL, USA; ^2^Sylvester Comprehensive Cancer Center, University of Miami Miller School of Medicine, Miami, FL, USA

**Keywords:** autoimmunity, antitumor, tumorigenesis, inflammation, cytokine

## Abstract

Cancer immunotherapy through manipulation of the immune system holds great potential for the treatment of human cancers. However, recent trials targeting the negative immune regulators cytotoxic T-lymphocyte antigen 4, programed death 1 (PD-1), and PD-1 receptor ligand (PD-L1) demonstrated that clinically significant antitumor responses were often associated with the induction of autoimmune toxicity. This finding suggests that the same immune mechanisms that elicit autoimmunity may also contribute to the destruction of tumors. Given the fact that the immunological identity of tumors might be largely an immunoprivileged self, autoimmunity may not represent a wholly undesirable outcome in the context of cancer immunotherapy. Rather, targeted killing of cancer cells and autoimmune damage to healthy tissues may be intricately linked through molecular mechanisms, in particular inflammatory cytokine signaling. On the other hand, since chronic inflammation is a well-recognized condition that promotes tumor development, it appears that autoimmunity can be a “double agent” in mediating either pro-tumor or antitumor effects. This review surveys the tumor-promoting and tumoricidal activities of several prominent cytokines: IFN-γ, TNF-α, TGF-β, IL-17, IL-23, IL-4, and IL-13, produced by three major subsets of T helper cells that interact with innate immune cells. Many of these cytokines exert divergent and seemingly contradictory effects on cancer development in different human and animal models, suggesting a high degree of context dependence in their functions. We hypothesize that these inflammatory cytokines could mediate a feedback loop of autoimmunity, antitumor immunity, and tumorigenesis. Understanding the diverse and paradoxical roles of cytokines from autoimmune responses in the setting of cancer will advance the long-term goal of improving cancer immunotherapy, while minimizing the hazards of immune-mediated tissue damage and the possibility of *de novo* tumorigenesis, through proper monitoring and preventive measures.

## Introduction: Paradoxical Relationships between Autoimmunity and Tumor

The process of inducing the immune system to selectively destroy tumor tissues *in vivo* faces numerous conceptual as well as practical hurdles. Foremost among these is the self-derived immunological identity of tumors (1, 2). The prevalence of self-antigen expression on cancer cells implies that many tumors will be protected from cytotoxic immune responses via intrinsic host mechanisms of self-tolerance. It is therefore evident that any biologic therapy capable of provoking a therapeutically useful antitumor immune response will carry some risk of off-target autoimmune toxicity. The resulting destruction of normal host tissues, besides contributing to morbidity and mortality in its own right, can potentially lead to *de novo* tumorigenesis by initiating chronic inflammation, which is a feature of premalignant states in numerous organs including breast, bladder, prostate, cervix, ovary, stomach, and lungs (3). The molecular sequence that links chronic inflammation to cancer development involves intricate and context-dependent interactions among differentiated tissue cells, immune cells, organ-specific stem cells, and other cell types present at the incipient tumor site (4). In light of these disparate outcomes, autoimmunity may be regarded as a “double agent” implicated in both immune-mediated tumor elimination and the cellular, genetic, and epigenetic changes that underlie carcinogenesis. Given the complexity and interconnections of the associated signaling networks, navigating this new therapeutic realm demands a formidable balancing act: any cancer treatment that seeks to modify immune system function must induce a degree of self-reactivity that leads to immune-mediated tumor killing while containing the destructive and cancer-promoting aspects of that self-reactivity within tolerable limits.

After decades of hard struggle in cancer immunotherapy, exciting opportunities have emerged, especially in monoclonal-antibody-based therapies designed to elicit antitumor immunity either through inhibition of negative immune system regulators or activation of costimulatory receptors (5). Remarkable benefits in patient survival have been demonstrated in clinical trials of novel monoclonal antibodies blocking immune checkpoints, including cytotoxic T-lymphocyte antigen 4 (CTLA4) (6), programed death 1 (PD-1) (7), and PD-1 receptor ligand (PD-L1) (8). In practice, however, many biologic therapies have fallen short of expectations in clinical trials, failing to deliver enhancements in disease-free or overall patient survival (9). A partial explanation for this disappointment is the context-dependent nature of immune signaling pathways themselves. In many cases, a given signal can exert diverse, and often opposing, effects on the progression of cancer depending on a variety of factors including the tissues involved, expression level of the signal molecule(s), tumor stage and antigenic profile, and host genetic background. Thus, there is a remarkable degree of overlap between the signaling mechanisms that mediate the desired outcome of tumor destruction, and those which fuel the detrimental processes of cancer development, tumor progression, and autoimmunity. Many cytokines with therapeutic potential have demonstrated these paradoxical effects, revealing both tumoricidal and tumor-promoting activities under different experimental conditions. The task of eliciting potent cytotoxic immune responses, while managing the concomitant dangers of autoimmunity, therefore requires detailed mechanistic knowledge of immune system signaling. This review will summarize the current understanding of the pro- and antitumor activities of several major cytokines (Table 1): interferon-gamma (IFN-γ); tumor necrosis factor-alpha (TNF-α); transforming growth factor-beta (TGF-β); the Th17 cytokines IL-17 and IL-23; and the Th2 cytokines IL-4 and IL-13. Concluding remarks will address a hypothetical loop of autoimmunity-mediated antitumor immunity, leading to further induction of autoimmune responses, inflammatory cytokine signaling, and tumor promotion, including potential *de novo* tumorigenesis in solid organs such as the gastrointestinal tract with demonstrated susceptibility to inflammatory carcinogenesis.

**Table 1:** **Anti- and pro-tumor activities of selected cytokines**.

Cytokine	Antitumor activities	Pro-tumor activities
IFN-γ	Required for Th1 differentiation, effective cytotoxic antitumor immune responses, and transplanted tumor rejection induced by bacterial endotoxin (10); mediates immune surveillance against spontaneous tumor development (11); enhances tumor antigenicity by upregulating expression of antigen presentation machinery (12); has immunosuppressive functions that can restrain chronic inflammation in certain contexts (13, 14)	Mediates chronic inflammation in gastric epithelium (15, 16); protects tumor cells from CTL lysis by altering their surface MHC expression (17, 18)
TNF-α	Mediates transplanted tumor rejection induced by bacterial endotoxin (19); local administration damages tumor vasculature, and has been used in isolated limb perfusion to treat melanomas and soft tissue sarcomas (20)	Mediates chronic inflammation, oxidative stress, and genomic damage, promoting malignant transformation in various organs (21–23); activates growth and survival-promoting pathways that drive angiogenesis, proliferation, invasion, and metastasis in established tumors (24, 25)
TGF-β	Regulates the cell cycle and inhibits proliferation in stromal and epithelial tissues (26, 27); restrains chronic inflammation by opposing Th1 differentiation and downregulating IFN-γ production by NK cells and DCs (28–30)	Induces tolerant, cancer-promoting phenotypes in tumor-associated macrophages (M2) and neutrophils (31); promotes differentiation and recruitment of immunosuppressive Treg cells in the tumor microenvironment (32, 33); promotes angiogenesis, invasion, and metastasis of cancer cells (34); may suppress antitumor immunity by inhibiting Th1 signaling (35)
IL-17/IL-23	May promote IFN-γ secretion and Th1 differentiation (36, 37); possible mediator of innate and adaptive antitumor immunity through interactions with the Th1 signaling axis (38–41), or direct cytotoxicity of Th17 cells (42)	Mediates chronic inflammation in the liver (43–46); drives differentiation and expansion of Th17 cells in the tumor microenvironment, which has been shown to favor disease progression and suppress antitumor immunity (47, 48); activates proliferative and survival-promoting pathways in cancer cells (49–51)
IL-4/IL-13	Poorly characterized	Drives differentiation of tolerogenic CD4+ Th2 cells in the tumor microenvironment (52); induces cancer-promoting phenotypes in tumor-associated macrophages (M2) and DCs (31, 53); IL-4 mediates survival and proliferation of cancer stem cells (54–56); IL-13Rα2 signaling promotes tumor invasion and metastasis (57–59)

## Anti- and Pro-Tumor Roles of Autoimmunity Could be Mediated through Inflammatory Cytokines of Th Cells

The pathological correlation between inflammation and cancer has been known to clinical science for 150 years (60). However, it is only in recent times that our understanding of the immune system has become sophisticated enough to yield practical applications in the realm of cancer biology. For example, ample evidence has been gathered for the roles of inflammatory signals derived from innate immune response in promoting tumor growth and progression [for recent reviews, please see Ref. (4, 61, 62)]. There is emerging evidence for a tumor-promoting role by inflammatory effectors from the adaptive immune system, for example, in the TRAMP model of prostate cancer initiated with transgenic expression of an oncogene (the SV40 large T antigen) (63, 64), or implanted model of melanoma (65). Given the long-lasting nature of memory responses that is characteristic of adaptive immunity and the potency of autoimmune memory T cells [for review see Ref. (66)], one might speculate that inflammatory signals originated from the adaptive immune system, compared to their innate-derived counterparts, might sustain a longer effect, regardless of their pro- or antitumor nature. However, we should also emphasize that in a complex *in vivo* setting of immune responses, it is likely that the intimate interaction between innate and adaptive immune cells determines an immunological outcome (67–69). Nevertheless, definitive evidence remains to be gathered to show whether an inflammatory signal(s), at a physiologically relevant setting, can initiate *de novo* tumorigenesis.

Numerous parameters of immune system function have been correlated with clinical outcomes in cancer patients, including the cellular composition of tumor inflammatory infiltrate (70), expression of pro- and anti-apoptotic genes in circulating PBMCs (71), and cytokine profiles measured in peripheral blood (72). It has long been recognized that the same inflammatory cytokine may play a prominent role in either tumor killing or cancer promotion. Although the original stimuli of inflammatory cytokine production are unknown in most cancer settings, given the largely “self” constituents of tumors, we reasoned that autoimmune T cells, especially the three main T helper subsets, Th1, Th2, and Th17, could be a major source of these cytokines, or play a major role in driving other immune cells to produce them.

Early experimental models offered hope that the induction of selective antitumor immune responses, even those mediated by CTL recognition of self-antigens, was possible without clinically significant autoimmunity. One study reported the eradication of established p53-overexpressing tumor cells in mice, achieved through adoptive transfer of a clone of cytotoxic T cells, which recognized wild-type p53. No autoimmune damage was observed in normal tissues, despite the p53+/+ genotype of the treated mice (73). *In vitro* assays also suggested that epitope-specific CTL clones had the capacity to initiate cytotoxic immune responses against non-mutated, tumor-associated p53, while simultaneously avoiding reactivity with the same antigen when endogenously expressed (74). Other experiments used transgenic models to search for evidence of autoimmune pathology in an organ-specific fashion. In one study, transgenic mice were engineered to express low levels of Friend murine leukemia virus envelope protein (FMuLV) from an immunoglobulin promoter; adoptive transfer of FMuLV-specific T cells mediated complete destruction of leukemia tumor cells, without concurrent autoimmunity. Lymphoid tissues in the treated mice were unharmed, despite the fact that they expressed the targeted cancer-associated antigen in a “self” context (75). Another study employed transgenic mice with tissue-specific expression of influenza virus hemagglutinin (HA) in pancreatic islet β cells. Administration of an anti-HA vaccine to these animals produced CTL-mediated rejection of renal carcinoma cells, which had also been genetically engineered to express HA. Prior to vaccination, the immune systems of the transgenic mice exhibited HA self-tolerance, and supported growth of implanted tumors expressing this antigen. Remarkably, in addition to tumor rejection, immunohistologic analysis of treated mice showed intact structure and function in the pancreatic islets (76).

Despite these favorable preclinical results, most types of cancer immunotherapy tested in human patients have revealed serious and persistent risks of treatment-associated autoimmunity. Reported manifestations include vitiligo, uveitis, psoriasis, and colitis, with potential consequences ranging from cosmetic complaints to permanent disability and death (77). However, it has been suggested that autoimmunity, in addition to mediating these adverse events, makes an essential contribution to antitumor immunity. Indeed, it may be the case that effective cancer immunotherapy requires a significant degree of self-reactivity, since antitumor immune responses must surmount both the preexisting tolerance to self-derived tumor antigens and immunosuppressive signals from the tumor microenvironment (1, 77). For some cancer immunotherapies, such as blockade of the inhibitory T cell signaling molecule CTLA4, autoimmune toxicity shows a positive correlation with therapeutic response (78). Meanwhile, antitumor vaccines, probably the category of cancer immunotherapy least associated with severe autoimmunity in human studies (77), have generally failed to show therapeutic efficacy in large-scale Phase 3 trials (79). Thus, it appears that the future of cancer immunotherapy will not feature the elimination of autoimmunity, which is unlikely to be feasible or wholly desirable. Rather, the cytokines that mediate autoinflammation can be harnessed as effective agents in tumor destruction, if their toxic and tumor-promoting potentials are understood and judiciously managed.

### IFN-γ

Interferons (IFNs) are a family of cytokines that bind to cell-surface receptors and mediate numerous functions related to pathogen defenses, immune function, cell survival and differentiation, and angiogenesis (12, 80). Type I interferons are broadly expressed (80), while IFN-γ, the sole member of the Type II interferon family, is produced mainly by T lymphocytes, NKT cells, and natural killer (NK) cells as a crucial component of the inflammatory response (10). IFN-γ is integral to the development of Th1 adaptive immunity, by maintaining IL-12 signaling in CD4^+^ T lymphocytes while simultaneously suppressing Th2 differentiation (10). Murine models support a range of anti-carcinogenic properties for IFN-γ (11). Antibody-mediated suppression of IFN-γ signaling has revealed that this cytokine is required for LPS-stimulated rejection of transplantable tumors in mice. IFN-γ receptor knockout mice (IFN-γR1^−/−^) exhibit susceptibility to both spontaneous and chemically induced tumors, in addition to poor resistance to a variety of pathogens (10, 11). IFN-γ is a powerful inducer of Class I and Class II antigen presentation machinery, suggesting its primary importance in the production of specific antitumor immune responses (12). *In vitro* assays have also revealed pro-apoptotic, antiangiogenic, and antiproliferative effects (10–12, 81). Indeed, reduced IFN-γ production has been observed in a variety of human malignancies, including melanoma, gastric cancer, lung cancer, glioblastoma, nasopharyngeal carcinoma, colorectal cancer, and head and neck cancer (72). Certain human cancers, notably melanoma, are known to develop resistance to IFN-γ through various mutations in downstream molecules in the IFN signal transduction pathway, such as JAK and STAT1 (12, 81).

Despite this promising body of experimental evidence, anticancer therapy with exogenous IFN-γ has generated mixed results. A 2003 clinical trial in malignant melanoma patients showed no positive responses to intratumoral injections of IFN-γ (82). A Phase 3 trial for ovarian cancer and peritoneal carcinoma was prematurely ended in 2006, due to decreased survival and more frequent adverse events in patients treated with subcutaneous IFN-γ, as compared to conventional platinum-based chemotherapy (82). To understand why this cytokine has not demonstrated the broad therapeutic efficacy that prior data would suggest, it is necessary to examine the dual nature of the inflammatory process. While inflammation can promote the cell-mediated destruction of tumor cells, its chronic forms may lead to pathological changes that promote cancer development in a variety of tissues. These changes include accumulation of reactive oxygen species, epithelial hyperplasia, extracellular matrix generation, and angiogenesis (3, 83, 84). Besides mediating anticancer immunity, IFN-γ is a major player in chronic inflammation, as illustrated by its contributions to Th1-driven autoimmune disease. In clinical trials, anti-IFN-γ antibodies have been useful in treating a variety of inflammatory disorders, including rheumatoid arthritis, Crohn’s disease, corneal transplant rejection, and skin diseases such as vitiligo and alopecia areata (82, 85, 86).

The interplay between IFN-γ signaling, aberrant chronic inflammation, and neoplastic disease has been explored in the pathogenesis of gastric cancer, one of the classic models of human malignancy precipitated by chronic inflammation (87–90). With regard to gastric carcinogenesis, the role of Th subsets would also perhaps be best surveyed while keeping in mind the robust evidence of innate immunity in gastric cancer development (91–93). In the natural course of this disease, infection with the bacterium *Helicobacter pylori* during childhood precipitates a chronic inflammatory response which, in a small portion of patients (<5%), progresses to malignancy decades in the future (84, 94). Studies of mice infected with the related pathogen *Helicobacter felis* showed that a Th1-biased inflammatory response is involved in gastric cancer development (94). Knockout of the transcription factor T-bet, which controls commitment to the Th1 lineage (95), curtailed gastric tumorigenesis induced in mice infected with *H. felis* (15). A recent experiment employed a murine model that directly implicates IFN-γ in this course of preneoplastic change. The transgenic mice, which were engineered to overexpress IFN-γ from a stomach-specific, H/K ATPase β promoter, exhibited a prominent inflammatory infiltrate along with accelerated histological changes characteristic of a premalignant phenotype: metaplasia, loss of parietal and chief cells, gastric gland atrophy, and dysplasia beginning as early as 3 months of age (16).

IFN-γ can thus be seen as an essential mediator of both immune-mediated tumor rejection and the destructive chronic inflammation that precedes malignant transformation. There is, however, another vital dimension to this cytokine’s effects, that of a master regulator which restrains inflammation in a variety of contexts. In two key murine model systems of Th1-driven autoimmunity, collagen-induced arthritis (CIA) and experimental autoimmune encephalitis (EAE), it has been established that abrogation of IFN-γ signaling through monoclonal antibody therapy or genetic knockout produces a seemingly paradoxical increase in disease susceptibility and severity (13, 14). The mechanisms for this apparent downregulation of the inflammatory response are not yet clear, but suppression of pro-inflammatory Th17 cytokines, induction of T cell apoptosis, and enhanced Treg cell differentiation appear to play prominent roles (13, 14, 96). Another mechanism of IFN-γ mediated immunosuppression is suggested by its ability to alter tumor cell MHC presentation in a manner that *decreases* tumor antigenicity and protects tumor cells from CTL killing. One *in vitro* experiment demonstrated that IFN-γ could protect ovarian carcinoma cell lines from CTL-mediated lysis by upregulating HLA-E on cancer cells, which engaged the inhibitory CD94/NKG2A receptor on CD8^+^ effector T cells (18). A similar cancer-promoting role for IFN-γ was revealed in a mouse model of melanoma, where tumor protection from CTL killing was associated with IFN-γ-stimulated upregulation of non-cognate MHC-I molecules (17).

Given the evidence above, IFN-γ-mediated downregulation of the inflammatory response can be regarded as an obstacle to effective cancer immunotherapy. However, it remains an important physiological mechanism for preserving tolerance to self-antigens and protecting tissues from the damaging effects of autoimmunity (80, 97). In keeping with this fact, experimental data also suggest that IFN-γ mediated suppression of inflammation may be protective in certain contexts. One recent experiment employed a transgenic mouse line engineered to overexpress IFN-γ in a stomach-specific manner, using the same H/K ATPase β promoter as Syu et al. (16). In this case, however, no spontaneous gastritis or metaplasia occurred (98). Moreover, IFN-γ was reported to be protective against gastric dysplasia produced by either *H. felis* infection or stomach-specific overexpression of the cytokine IL-1β (a well-established mediator of gastric carcinogenesis). IFN-γ was also shown to inhibit proliferation of gastric epithelial cells, enhance autophagy in a manner recognized as protective against tumor development (99), and diminish expression of pro-inflammatory Th1 and Th17 cytokines (98). As noted by Syu et al. (16), the seemingly contradictory results of these two studies can most likely be explained by differing degrees of overexpression. These findings underscore the challenges of discerning which aspect of IFN-γ will prevail in a particular patient, tumor type, and stage of malignancy. Attempts to broaden the therapeutic use of this cytokine must take into account these intricate and context-dependent multidirectional effects.

### TNF-α

Activated macrophages and T lymphocytes produce TNF-α in response to pro-inflammatory stimuli. This cytokine stimulates inflammation through multiple mechanisms, including recruitment of neutrophils and monocytes and induction of cell adhesion molecule expression on the endothelial surface (72, 100). TNF-α is involved in the classical pathway of macrophage activation (M1), which plays a central role in immune defenses against tumors and intracellular parasites (31). Although usually undetectable in the tissues and circulation of healthy people, TNF-α exerts important effects on immune homeostasis during states of disease. It is known, for instance, that systemic TNF-α mediates the wasting observed in humans and animals afflicted with chronic illness (24, 100, 101). As with IFN-γ, aberrant TNF-α signaling is associated with a variety of autoimmune disorders. Five TNF inhibitors have been approved for clinical use in the United States for the treatment of inflammatory illnesses such as Crohn’s disease, ankylosing spondylitis, and rheumatoid arthritis (100, 102).

The observation that the human body’s response to infection is capable of producing regression of tumors has been recorded by clinicians since at least the eighteenth century (103). It was not until 1975, however, that TNF-α was identified as one of the principal mediators of this anticancer immune response. The name “tumor necrosis factor” reflects early experiments, which demonstrated this cytokine’s capacity to induce hemorrhagic necrosis of subcutaneously transplanted sarcomas, leukemias, and mastocytomas in mice whose immune systems were primed by exposure to bacterial endotoxin (19). However, 38 years of data subsequent to this discovery have not fulfilled the early promise of TNF-α as a safe, potent, or selective tumoricidal agent. Unlike mice with abrogated IFN-γ signaling, TNF-α-knockout animals do not develop spontaneous tumors, and peritoneal tumors transplanted into them do not show accelerated growth (104). These knockout animals have also shown an unexpected resistance to chemically induced skin tumors (105). Indeed, there seem to be numerous contexts in which TNF-α signaling helps to initiate carcinogenesis and sustain tumor growth. In obese patients, TNF-α is suggested to be a key mediator of cancer-promoting inflammation in various organs, including the pancreas (22), colon (21), and liver (23). Picogram amounts of TNF-α are constitutively secreted by many tumor types, and appear to stimulate cancer growth, although the underlying signaling mechanisms are not completely understood (24, 106). The pro-carcinogenic functions of TNF-α appear to be mediated predominantly through downstream activation of the proliferative and survival-promoting pathways NF-κB and AP-1 (20, 21, 100, 104). Elevated serum concentrations of TNF-α have been described as a clinical feature of eight independent cancer types (72). A direct link between the pro-inflammatory effects of TNF-α and carcinogenesis can be seen by returning to the example of gastric cancer. The presence of a specific, inflammation-associated single nucleotide polymorphism in the *TNF-A* gene has been found to increase the odds ratio of non-cardia gastric cancer (83). The importance of this association has been further established by examining the genome of the initiating pathogen: *H. pylori* harbors the Tipα gene family, whose members are known to induce high levels of TNF-α expression in the gastric epithelium (24, 106). Experimental transfection of a transformed BALB/3T3 cell line with *H. pylori* genes revealed that tumor progression was dependent on Tipα-induced production of TNF-α (106).

TNF-α has also been implicated as a disease promoter in hepatocellular carcinoma (HCC), from chronic inflammation to tumor progression including invasion, metastasis, and angiogenesis in established HCC tumors (107). *In vitro* studies of numerous cell types, including murine hepatocytes, have demonstrated that TNF-α-mediated inflammation leads to an increase in markers of oxidative stress, which in turn can lead to genomic damage (108–111). Moreover, the development of obesity-induced hepatosteatosis and steatohepatitis in mice is dependent on signaling by TNF-α, along with IL-6 (21). In the Mdr2-knockout mouse, an experimental model of inflammation-induced HCC, the spontaneous development of hepatic malignancy is dependent on TNF signaling, and can be attenuated through downstream inhibition of NF-κB (23). Overall, experiments on HCC and many other tumor types have established that TNF-α is intimately involved in all aspects of cancer development, including transformation, proliferation, angiogenesis, invasion, and metastasis (25).

Owing to a high frequency of inflammation-related adverse events, the therapeutic applications of exogenous TNF-α in cancer have been quite limited (24). Localized TNF-α infusion has demonstrated antitumor efficacy against melanomas and soft tissue sarcomas in human patients. In this context, high concentrations of TNF-α were shown to induce hyperpermeability and structural disruption in tumor vasculature, thus promoting tumor necrosis and enhancing the efficacy of traditional cytostatic drugs. However, this intervention was aimed at limb sparing and had no impact on patient survival (20). Given the broad range of cancer-promoting activities of TNF-α, blockade of its signaling remains a tempting therapeutic approach. Unfortunately, the fundamental role of TNF-α in pathogen defense represents a formidable obstacle to implementation (24). The feasibility of modifying TNF-α signaling without triggering destructive autoimmunity on one hand, or vulnerability to opportunistic infections on the other, remains to be seen.

### TGF-β

Transforming growth factor-beta mediates a vast array of functions related to wound healing, immune responses, cell proliferation and differentiation, and carcinogenesis, via receptors expressed on nearly all human cells. It plays a crucial role in T cell tolerance (112). Its classical signal transducers are transcription factors known as Smads, which combine with each other as well as additional cofactors to form a variety of DNA-binding complexes. These intricate assemblies of transcriptional regulators allow TGF-β to implement a versatile, yet precisely controlled, range of downstream effects. Additional “Smad-independent” signaling pathways are known to further augment this functional repertoire (27, 29, 34, 35). This cytokine has been extensively characterized as a negative regulator of immune responses, with anti-inflammatory, antiproliferative, and immunosuppressive activities (27, 29, 34, 35). Thus, TGF-β helps restrain the destructive effects of uncontrolled inflammation and proliferation that might otherwise occur in the context of infection or tissue damage. Mouse models reveal that knockout of TGF-β or its receptor produces a phenotype characterized by lethal autoimmune disease (113, 114). One recent study featured a murine model with the TGF-β receptor gene deleted in stromal fibroblasts. The transgenic animals experienced an excessive, aberrant inflammatory response in adjacent epithelial tissue, characterized by molecular markers of DNA damage, oxidative stress, cell cycle dysregulation, and death from invasive squamous cell carcinoma by the age of 7 weeks (26). Other models have examined the specific effects of TGF-β silencing in the innate immune and T cell compartments. One experiment blockaded TGF-β signaling in mouse NK cells through transgenic expression of a dominant negative receptor. NK cells were more numerous in lymphatic tissues of the transgenic mice, and showed enhanced secretion of IFN-γ. Consistent with the Th1-promoting effects of IFN-γ signaling, transgenic mice also demonstrated an enhanced Th1 inflammatory response, which protected against infection with cutaneous Leishmaniasis (28). These effects suggest the importance of TGF-β in maintaining NK cell homeostasis, as well as downregulating Th1 differentiation. Another study reported a dendritic cell-specific deletion of the TGF-β receptor gene, which resulted in multiorgan autoimmunity, a pro-inflammatory DC phenotype characterized by IFN-γ overproduction, and reduced Foxp3 expression in Treg cells (30). Accumulating evidence suggests that TGF-β is directly involved in many aspects of T cell homeostasis, including differentiation of Treg cells and CD8^+^ effectors, maintenance of peripheral tolerance, and preservation of naïve T cell populations (112). The precise molecular mechanisms behind these effects are largely unresolved, and represent an area of active investigation.

In the early stages of carcinogenesis, TGF-β is known to suppress tumor growth through induction of cell cycle inhibitors and promotion of apoptosis. However, in many advanced cancers, paracrine and autocrine TGF-β signaling drives tumor progression and metastasis. TGF-β is produced in large quantities by a variety of human cancers, to the extent that it is arguably the most ubiquitous immunosuppressive mediator in cancer progression (34). Elevated systemic levels of TGF-β have been reported in breast cancer, lung cancer, pancreatic cancer, glioblastoma multiforme, colorectal carcinoma, HCC, renal cell carcinoma, and gastric carcinoma (72). TGF-β is a crucial inducer of pro-tumor phenotypes in both tumor-associated macrophages (TAMs) and tumor-associated neutrophils (TANs), leading to cancer cell proliferation and the curtailing of antitumor immune responses (31). The capacity of TGF-β to promote the differentiation of Treg cells appears to be highly deleterious in this context, since Treg cells are key inducers of immune tolerance in the tumor microenvironment. The association between TGF-β signaling and Treg cell recruitment has been experimentally demonstrated in a lung cancer cell line (115), as well as in animal models of pancreatic cancer (32) and HCC (33). One key observation from these studies is that a tolerogenic immune microenvironment is not exclusively the result of tumor-secreted cytokines. Rather, it requires elaboration of TGF-β and other anti-inflammatory signals from immune cell populations such as dendritic cells and TAMs.

Several other mechanisms are believed to underlie the pro-carcinogenic role of TGF-β, including enhanced extracellular matrix formation, cytoskeletal rearrangements to facilitate epithelial-to-mesenchymal transition (EMT), angiogenesis, and cell cycle dysregulation (27, 29, 34, 35). In a clinical context, TGF-β serves as a marker of metastasis and poor prognosis for many malignancies (35, 72). The transition of TGF-β from a protective role early in tumor development to a tumor-promoting one in more advanced disease appears to be a watershed moment in many cancers, reflecting global derangement of signal transduction through genetic and epigenetic mechanisms. Indeed, *in vitro* experiments have linked specific forms of oncogenic transformation to alterations in TGF-β responsiveness. One study found that engineered overexpression of HER2 in mesenchymal human breast cancer cells caused a loss of sensitivity to the antiproliferative effects of TGF-β (116). Another reported that loss of TGF-β growth inhibition correlated with the loss of c-myc downregulation in ovarian carcinoma cells (117).

The prevalence of TGF-β overexpression across a broad range of human malignancies has made this cytokine a tantalizing therapeutic target. Four classes of TGF-β inhibiting molecules have already been tested in clinical trials, with responses that generally fell short of hopes (118). At least one class of TGF-β inhibitor has also shown the capacity to elicit biochemical resistance in mouse models (119). This observation, coupled with the integral roles of TGF-β in wound healing and tissue homeostasis, suggests that long-term inhibition of TGF-β signaling may be a dangerous prospect. Rather, it has been suggested that TGF-β inhibition will find its first clinical applications as part of a combined drug regimen, administered to cancer patients over relatively brief spans of time to minimize resistance and adverse events (118).

### IL-17 and IL-23

The Th17 subset of CD4^+^ T cells has added a new dimension of complexity to the Th1/Th2 paradigm since its initial discovery in 2005. Extensive studies in murine models have implicated this T cell population in a number of pro-inflammatory functions, including the pathogenesis of autoimmune diseases of the brain (EAE) and joints (CIA), mediated by the characteristic cytokine IL-17 (36, 120–122). Knockout mice have also revealed an important role for IL-17 signaling in the defense of mucosal surfaces against a variety of bacterial and fungal pathogens, including *Staphylococcus aureus, Klebsiella pneumoniae, Citrobacter rodentium*, and *Candida albicans* (36, 120). Th17 cells also engage in physiologically important interactions with key cytokines of the Th1 immune system. One attribute, noted in the initial characterization of this helper T cell subset, is the inhibitory effect of IFN-γ on Th17 differentiation and production of IL-17 (123, 124). Meanwhile, IL-23, a cytokine that is essential to sustain the survival and proliferation of Th17 populations, appears to participate in divergent regulatory pathways with the Th1-associated cytokine IL-12, whose release is promoted by IFN-γ (125–127). The dichotomous signaling of IL-12/IL-23 is especially intriguing, as these two cytokines share a common subunit: IL-12 is formed through the covalent linkage of p40 and p35 subunits, whereas IL-23 is a combination of p40 and p19 (128). TGF-β signaling also appears to make a major contribution to Th17 differentiation, although the precise nature and mechanism of this association remains a subject of intense controversy (122, 129).

Th17 signaling through the canonical IL-23/IL-17 pathway has been shown to contribute to cancer development in a variety of experimental contexts. During the preneoplastic stage, excessive Th17-mediated signaling is a probable contributor to the chronic inflammation that can precipitate cancer in a variety of tissues. In the case of Hepatitis B-induced inflammation, evidence from human and animal experiments suggests that Th17, not Th1, signaling is the primary mechanism underlying liver immunopathology and eventual malignant transformation to HCC (43–46). Th17 cells have been discovered in human tumors in many different organs, and a growing body of evidence suggests that their presence, like that of immunosuppressive Treg cells, may be a general feature of the tumor microenvironment (37, 121). One comprehensive examination of gene expression profiles in human cancers revealed overexpression of IL-23 in the vast majority of human carcinomas, when compared to expression profiles of adjacent, non-cancerous tissue (130). A recent quantitative PCR analysis revealed statistically significant IL-23 upregulation across a panel of non-small cell lung carcinoma (NSCLC) patient samples, in comparison to matched normal tissue controls (49). In mouse models, knockout of IL-23 conferred resistance to both chemically induced and transplanted epithelial tumors, whereas knockout of IL-12 produced the opposite effects (130). Moreover, there is mounting evidence that tumor cells produce chemokines in order to selectively recruit Th17 lymphocytes (121). In the 4T1 mouse model of breast cancer, Qian et al. reported that signaling via mammary tumor-derived prostaglandin E_2_ (PGE_2_) caused overexpression of IL-23 (but not IL-12) in the tumor microenvironment (48). This overexpression, in turn, was associated with expansion of Th17 cell populations in the tumor tissue, spleens, and peripheral blood of experimental animals (48). This discovery is likely to have significant implications in a variety of human cancers, since PGE_2_ is the most abundant prostanoid in epithelial cell tumors (127). In the same study, exposure of murine dendritic cells to tumor-conditioned medium enhanced their expression of the IL-23 subunit p19, and also reduced expression of p40 and the IL-12 subunit p35, in a dose-dependent manner (48). These changes collectively indicate a shift from Th1 to Th17 immune phenotype, which seems to be favorable for cancer persistence. As in the case of experimental models of TGF-β signaling, cells of the innate immune system were key participants in the generation of an immune microenvironment promoting carcinogenesis.

The pathways through which Th17 signaling exerts its tumor-promoting effects are not yet clear, but a variety of relevant observations have emerged. One probable mechanism is the stimulation of angiogenesis and cell proliferation: the p19 subunit of IL-23 is transcriptionally upregulated by growth-promoting signals from NF-κB and AP-1 (48). Administration of recombinant IL-23 has been shown to enhance proliferation in an IL-23 receptor-positive lung adenocarcinoma cell line (49, 51). One study reported IL-23-mediated enhancement of cancer growth and proliferation in cultures of human oral squamous cell carcinoma; in this case, IL-23 exposure was accompanied by enhanced nuclear translocation of NF-κB (50). Mouse models have also furnished valuable mechanistic clues. In one experiment, IL-17 knockout mice showed reduced growth of transplanted B16 melanoma and MB49 bladder carcinoma, whereas acceleration of tumor growth occurred with knockout of IFN-γ. In mice with both knockouts, tumor growth was reduced relative to WT controls (65). In the IFN-γ knockout mice, elevated concentrations of IL-17 were measured in tumor tissue compared to tumors in wild-type controls. Moreover, IL-17 was found to enhance signaling by the pro-survival, pro-angiogenic transcription factor Stat3 in both tumor and stromal cells (65). Another study reported similar results using receptor knockouts: IL-17 receptor-deficient mice showed diminished tumor growth of transplanted melanoma and lymphoma cell lines, while IFN-γ receptor knockout led to growth enhancement. The double-knockout genotype was also protective in comparison to WT controls (47). In addition, IL-17 receptor deficiency was correlated with increased tumor infiltration of CD8^+^ effector T cells, and decreased numbers of myeloid-derived suppressor cells (MDSCs), both favorable indications for the generation of antitumor immunity (47). Interestingly, these pro- and antitumor effects were successfully reproduced in wild-type mice through administration of either recombinant IL-17 (which accelerated tumor growth) or antibody-mediated IL-17 blockade (which suppressed it), hinting at the viability of anticancer therapies targeting this pathway (47). It is also notable that elimination of IL-17 signaling in both studies produced an antitumor effect that was potent enough to compensate for the concurrent loss of IFN-γ signaling, despite the role of IFN-γ as a potent mediator of Th1-driven antitumor immunity.

The complex and heterogeneous functions of the Th17 signaling axis, and their relationships to cancer progression, are only beginning to be elucidated. Despite the results mentioned above, numerous studies support the existence of antitumor Th17 effects, including those which may be mediated through signals other than IL-17 and IL-23 (121). For instance, subsets of Th17 cells are capable of producing IFN-γ, indicating possible cross-talk with the Th1 pathway of differentiation, as well as the ability to stimulate cytotoxic and tumoricidal immune responses (36, 37). In human pancreatic ductal adenocarcinomas, one recent study found correlation of protective benefit with activity of Th17 cells specific to α-enolase, a pancreatic tumor-associated antigen (42). Another recent study reported that a mouse model deficient in RORγt (a transcription factor required for Th17 differentiation) exhibited accelerated growth of transplanted melanoma tumors, along with a diminished percentage of Th1 CD4^+^ cells at the tumor site; this phenotype was rescued by adoptive transfer of Th17 cells, a portion of which began to produce measurable quantities of IFN-γ (41). One study of IL-17 knockout mice demonstrated increased susceptibility to lung melanoma. Subsequent treatment by Th17 adoptive transfer served to prevent tumor development by inducing a specific CD8^+^ antitumor response (40). It has also been found that systemic administration of high-dose IL-23 led to reduced tumor growth and prolonged survival in a mouse fibrosarcoma, due to Th1-mediated activation of cytotoxic T cells, helper T cells, and NK cells (38). Increased growth and lung metastasis of murine colon carcinoma has also been reported in IL-17 deficient animals, with corresponding reductions in IFN-γ^+^ NK cells and IFN-γ^+^ tumor-specific T cells (39).

Although characterized less than a decade ago, the Th17 lymphocyte population has already become the focus of a vast and diverse body of scientific literature. However, this expanded knowledge contains apparent contradictions, which will challenge the field of cancer immunology for years to come. As with the pathways and cytokines previously discussed, the tumor-related effects of IL-17 and IL-23 exhibit a high degree of context dependence. Some of these discordant results may therefore be attributable to the source of tumor cells, the tissue involved, the stage of cancer growth, the genetic background of the organism, and other features of the experimental model employed in a particular study. Meanwhile, our understanding of Th17 interactions with other elements of the immune system, including NK cells, antigen-presenting cells and other helper T cell subsets, remains incomplete. Experiments designed to address the cross-talk between Th17 cytokines and other branches of the immune system should help resolve some of the inconsistencies in their reported effects. While a variety of IL-17/IL-23 antagonists are currently being developed for the treatment of autoimmune diseases (131), safe and effective modification of Th17 signaling in cancer therapy will require a more thorough understanding of the forces which underlie Th17 differentiation, recruitment, and interaction with malignant cells.

### IL-4 and IL-13

The Th2 subset of CD4^+^ T cells plays an important physiological role in implementing immune defenses against helminths and other extracellular parasites. Th2-mediated responses include generation of high-affinity IgE antibodies, mucus overproduction, and heightened smooth muscle contractility, all of which function in the clearance of invasive multicellular organisms. However, aberrant and excessive Th2 activation also provides the foundations for allergic disease (132). The major cytokine responsible for differentiation of naïve CD4^+^ T cells into the Th2 phenotype is IL-4, while Th2 effector functions are mediated through a combination of IL-4, IL-13, and IL-5 (133). All three of these cytokines exert varied effects on cancer development, which remain an area of ongoing investigation. Th2 cytokines appear to be involved in shifting the immune response to forms favorable to tumor growth, particularly in the context of innate immunity. IL-4, IL-13, and IL-5 promote the differentiation of macrophages into an “M2” or alternatively activated form, which displays poor antigen-presenting capacity and local anti-inflammatory effects (31, 83, 134). M2 macrophages play a variety of physiological roles in tissue homeostasis, including wound healing, extracellular matrix remodeling, and scavenging of debris (132, 134). This M2 phenotype contrasts with the classically activated (M1) macrophage, which is specialized for the production of pro-inflammatory cytokines (e.g., IFN-γ), cytotoxic immune responses, and efficient destruction of phagocytosed microbes (132, 134). In the context of cancer, M2 polarization of TAMs is associated with suppression of antitumor immune responses, disease progression, and poor prognosis (31, 83). Moreover, it has been established that tumors are capable of producing Th2 cytokines in order to bias innate and adaptive immune responses toward this more favorable phenotype (134). A study of pancreatic cancer patients demonstrated that tumor-produced cytokines (TNF and IL-1β) triggered activation of a Th2 phenotype in cancer-associated fibroblasts, dendritic cells, and naïve CD4^+^ T cells. Moreover, the ratio of Th2:Th1 CD4^+^ T lymphocytes present at the tumor site was negatively correlated with patient survival (52). In a humanized mouse model implanted with human breast carcinoma, Th2 cytokine expression was detected in both cancer cells and tumor-promoting CD4^+^ T cells within the tumor microenvironment. Dendritic cells isolated from these tumors also potently induced Th2 cytokine secretion from naïve CD4^+^ T cells *in vitro*, suggesting that tumor growth is facilitated by a complex network of Th2 paracrine signals (53).

The Th2 cytokine IL-4 is capable of signaling through two distinct cell-surface receptor complexes. The Type I receptor, found on cells of hematopoietic stem cell origin, is composed of IL-4Rα and the common gamma chain γc. The Type II receptor, expressed on cells of non-hematopoietic origin, contains IL-4Rα and IL-13Rα1, and also binds the cytokine IL-13 (135). IL-13Rα2 is a second type of IL-13 binding receptor, whose physiological role remains uncertain. The receptor bears structural similarities to IL-13Rα1, but is expressed in two forms: as soluble IL-13Rα2, and as a transmembrane protein, which interacts with a number of signal transduction pathways (54). IL-4 and IL-13 exert both overlapping and distinct physiological effects by binding these receptors, whose structure and function have been extensively studied as potential therapeutic targets in asthma and other allergic diseases (135, 136). Emerging evidence indicates that these receptors can influence cancer development through pathways other than macrophage polarization, although the molecular details of this process are only starting to become clear.

The Type II IL-4/IL-13 receptor has been found to be overexpressed in a variety of epithelial tumors, and treatment of cancer cell lines with IL-4 is associated with pro-proliferative and anti-apoptotic effects (54, 137). The effects of IL-4 signaling have been studied extensively in the development of colon cancer: one study found pro-proliferative effects of IL-4Rα signaling in mouse colon tumors, as well as human and mouse colon adenocarcinoma cell lines examined *in vitro* (55). Of particular interest is the relationship between intestinal malignancies and multipotent stem cells, which have been identified in recent experiments as an integral driving force in the growth of both premalignant adenomas and established tumors (138, 139). It now appears that IL-4 signaling is vital to the functioning of at least some of these tumorigenic stem cells. Research on colon cancer has identified a subset of tumor cells with a CD133^+^ stem-like phenotype, which was found to be necessary and sufficient for the establishment of transplanted human colon tumors in immunodeficient mice (56). In keeping with the cancer stem cell (CSC) hypothesis (4), these cells possess self-renewing capacity, an especially high resistance to death-promoting signals, and the ability to effect regeneration of the overall tumor mass (56, 140). One study revealed that resistance to drug-induced apoptosis in CD133^+^ colon cancer cells was mediated through increased production of IL-4 (56). Preliminary experiments suggest that this IL-4-mediated, pro-survival pathway may be a promising therapeutic target. In the same study, blockade with either IL-4 neutralizing antibody or a mutant, inhibitory form of IL-4 (IL-4DM) reduced the viability of CD133^+^ and CD133^−^ tumor cell cultures, while increasing the efficacy of cytotoxic treatment with standard chemotherapeutic agents: oxaliplatin, 5-fluorouracil, and the death receptor ligand TRAIL. IL-4 antagonism also enhanced the effectiveness and duration of chemotherapy response in mice bearing transplanted tumors, suggesting a role for combined therapy in the treatment of human disease (56). Both the *in vitro* and *in vivo* effects of IL-4 blockade were mediated by decreases in the anti-apoptotic molecules cFlip, Bcl-xL, and Ped (56, 140).

Another remarkable finding has been the role of IL-13Rα2 in models of cancer development, indicating signaling functions far beyond the previously suggested role of a decoy receptor (141). This receptor is known to be overexpressed in several human cancers. An immunohistochemical analysis of human tissues found IL-13Rα2 overexpression in 71% of pancreatic ductal adenocarcinoma samples in comparison to normal pancreas controls (142). Indeed, experiments in an orthotopic mouse model of pancreatic cancer suggest that IL-13Rα2 is an important mediator of the pro-tumor effects of IL-13, including activation of AP-1 growth signals, production of immunomodulatory cytokines such as TGF-β, and promotion of metastasis (58, 142). Similar IL-13Rα2-dependent effects have been demonstrated in other cancer models, including ovarian carcinoma (59), colorectal cancer (57), head and neck squamous cell carcinoma (143), and malignant glioma (144). As with IL-4, IL-13 signaling has been suggested as a possible target of anticancer therapy. To this end, recombinant cytotoxic proteins have been developed, which consisted of IL-4 or IL-13 joined to a mutant form of *Pseudomonas* exotoxin. These agents have been found to restrain tumor growth in numerous animal models (142–144). However, in a Phase 3 clinical trial, IL-13 *Pseudomonas* exotoxin failed to improve median survival time in patients with glioblastoma multiforme when compared to conventional chemotherapy (145). While this agent may still find use as an adjuvant therapy (146), the outcome suggests that the development of Th2-targeted treatments with robust antitumor efficacy will require further exploration.

Despite considerable progress in the field, many Th2-mediated influences on tumor development remain poorly characterized. For instance, populations of eosinophils and mast cells, crucial mediators of Th2-driven allergic responses, also contribute to the inflammatory infiltrate in numerous human tumors. However, experimental data exploring their effects are lacking, and conflicting results have been published regarding their impact on clinical prognosis (83). This ambiguity underscores the fearsomely complex, and incompletely understood, nature of signaling among discrete immune system components in the context of cancer development. Owing to these knowledge gaps, the possibility that an anti-Th2 intervention could impede antitumor immune responses *in vivo* cannot be prematurely dismissed. Systemic therapies to antagonize Type I or Type II receptors will also confront a high risk of adverse events, owing to the global effects of Th2 cytokines on immune homeostasis and other physiological functions. Nevertheless, a fascinating body of experimental and clinical data suggests that the pro-carcinogenic effects of IL-4 and IL-13 will remain a source of therapeutic interest for years to come.

## Potential Feedback between Autoinflammation and Tumor: Implications for Cancer Immunotherapy

**Figure 1:**
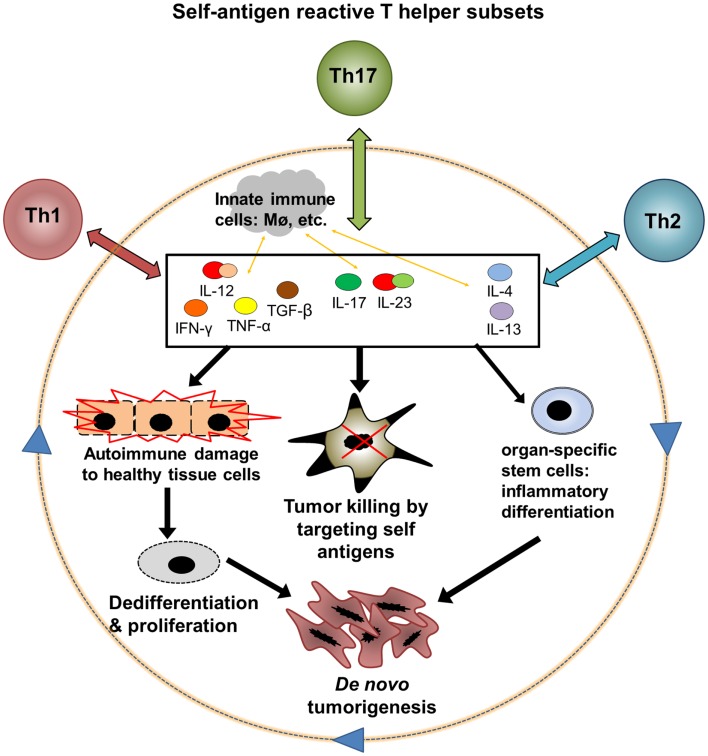
**A hypothetic feedback loop between autoimmunity, antitumor immunity, and inflammatory tumorigenesis**. Three major subsets of autoantigen reactive T helper cells, Th1, Th2, and Th17, could mediate both antitumor and pro-tumor effects through interaction with innate immune cells and CD8 T cell effectors. (1) Th1, Th2, and Th17 cells release cytokines after encountering autoantigens. These cytokines may exert direct and indirect effects on cell cycle regulation, apoptosis, and differentiation in both tumors and healthy tissues. (2) Cytokines can promote tumor killing by various mechanisms including stimulation of CD8 effectors, innate immune cell activation, and direct toxicity. (3) Inflammation in healthy tissues, particularly when chronic, could contribute to loss of antiproliferative signals and aberrant differentiation in normal cells, leading to a premalignant phenotype. Continued proliferation of these abnormal cells, with accumulation of DNA damage, leads to the emergence of cancer. (4) Chronic cytokine signaling may induce abnormal differentiation of organ-specific stem cells or other precursor cells and drive a cascade of cancer development. The feedback between autoinflammation and cancer may particularly affect solid organs such as the gastrointestinal tract that are demonstrated to be susceptible to inflammatory carcinogenesis.

Virtually all of the immune signaling pathways relevant to tumor biology play major physiological roles in the maintenance of self-tolerance and tissue homeostasis. Moreover, most well-characterized tumor-associated antigens are self-antigens, meaning that they are also expressed by normal cells in the course of growth and differentiation. Conversely, antigenic proteins expressed exclusively in cancerous tissue, such as viral products or mutated oncogenes, have been characterized in only a small number of tumor types. Overall, it seems that tumors, despite their aberrant characteristics, may remain antigenically “self” entities first and foremost (1, 2). In the clinical context, this suggests that useful antitumor immune responses elicited in patients may be functionally inseparable from those directed against healthy tissues. The seemingly inextricable association between off-target autoimmune damage to healthy tissues and antitumor immunity induced by effective cancer immunotherapy during recent clinical trials can be seen as supporting this hypothesis. Evidence from molecular studies in this regard and a large body of research on inflammation and cancer suggest a feedback loop of autoimmunity, antitumor immunity, inflammation, and *de novo* tumorigenesis that reinforces the remarkable entanglement between autoimmunity and cancer **(**Figure 1**)**. In particular, the persistence of antigens in most autoimmune conditions likely leads to the formation of a type of autoimmune memory cells called effector memory T (T_EM_) cells [for review see Ref. (66)], which could further perpetuate this feedback loop. Advances in not only experimental and clinical research, but also in computational biology tools for large datasets, will be needed to understand the complexity of molecular and cellular interactions in such chronic human disease settings (66, 147).

Along this line, one may argue that a combination of immunomodulation with conventional cancer therapies such as chemotherapy could be used to augment tumor-specific immune responses. Despite the severe immunosuppression produced by many standard chemotherapeutic agents, current data suggest that patients with chemotherapy-induced leukopenia retain a functional T cell compartment that is capable of mediating clinically significant antitumor immunity (148). Within solid tumors, it has been demonstrated that chemotherapy can deplete immune suppressor cells (MDSCs and Treg cells) in the tumor microenvironment, increase tumor antigenicity, and upregulate the expression of costimulatory molecules for CTL activation (79). All of these mechanisms point toward possible synergistic effects that could enhance the clinical efficacy of existing cancer immunotherapies, including agents that have shown meager benefits when administered alone. Indeed, synergy between cancer vaccines and chemotherapy has already been demonstrated in studies of advanced small-cell lung cancer and follicular B-cell lymphoma (149).

Immunological research continues to produce crucial mechanistic insights into the tumor-related effects of major cytokines. For instance, a recent murine experimental model has demonstrated that IFN-γ and TNF-α produced by Th1 cells are capable of inducing prolonged senescence in pancreatic tumors, by inducing expression of the transcription factors JUNB and INK4A (150). The identification of specific signaling pathways for tumor cell growth inhibition and apoptosis heralds a new and improved generation of cytokine-based therapies. More broadly, this knowledge may eventually enable a combinatorial approach to cancer immunotherapy, in which multiple treatments can be jointly administered to yield superior therapeutic outcomes. The current field of cancer immunotherapy is divided between treatments that encourage global activation of cytotoxic immune responses, such as exogenous cytokines and antibodies targeting T cell-inhibitory signals (e.g., anti-PD-1, anti-PD-L1, anti-CTLA4), and treatments based on tumor antigens, which aim to stimulate destruction of cancerous tissues by engaging a specific population of tumor-reactive CTLs (e.g., cancer vaccines and autologous T cell transfers). With further advancement in the clinical and investigative realms, it may become possible for these two approaches to complement each other within the same patient. A cancer vaccine that produces a meager antitumor response *in vivo* could have enhanced efficacy when administered alongside a treatment that elicits global T cell activation, such as CTLA4 blockade. Moreover, the systemic autoimmune toxicity produced by these broad-acting treatments might be mitigated if a lower dose was combined with a cancer vaccine or other antigen-focused immune stimulus. Adding to the possibilities, multiple immunomodulatory agents from either treatment category could theoretically be combined (e.g., anti-PD-1 in combination with one or more exogenous cytokines) to provide unprecedented control over the targeting, intensity, and duration of the induced immune response. The potential advantages of a combined regimen are already supported by data from numerous preclinical models (5). The true capabilities of cancer immunotherapy may only be realized once multiple treatments can be synthesized into a therapeutic strategy tailored to the pathological and molecular characteristics of every patient’s disease. Although the harmful clinical sequelae of autoimmunity may never be banished entirely, this integrative approach has the potential to harness its tumoricidal functions better than any single agent administered in isolation.

In summary, clinical data suggest that both the anticipated benefits of cancer immunotherapy and its associated adverse events share autoimmunity as a common originating process. Thus autoimmunity, regarded until recently as a “side effect” of cancer immunotherapy, may be more properly considered a correlate of antitumor immunity, or even more appropriately as an antitumor effector in its own right. Despite the lopsided benefit versus risk ratio in cancer immunotherapies that succeed in providing substantial survival benefit, it should be noted that autoimmune damage to healthy tissues is a justifiably dreaded cause of morbidity and mortality in patients receiving these treatments. This hazard may be even more dire if one considers that an immunosuppressive microenvironment effectively designates tumors as an immunoprivileged self, which is more resistant to immune targeting than its healthy counterpart (1, 2). Furthermore, the calculation of benefit versus risk must account for the possibility that inflammatory signals arising from therapeutically induced autoimmunity may ultimately contribute to *de novo* tumorigenesis in the clinical setting. Therefore, achieving optimal benefit of cancer immunotherapies awaits advances in tumor-specific targeting, either by site or by unique antigens, coupled with proper monitoring and prevention of potentially catastrophic autoimmune damage or long-term risks of *de novo* tumorigenesis.

## Conflict of Interest Statement

The authors declare that the research was conducted in the absence of any commercial or financial relationships that could be construed as a potential conflict of interest.

## References

[1] Miska J Bas E Devarajan P Chen Z Autoimmunity-mediated antitumor immunity: tumor as an immunoprivileged self. Eur J Immunol (2012) 42:2584–2596 10.1002/eji.201242590 22777737

[2] Miska J Devarajan P Chen Z The immunological identity of tumor: self implications. Oncoimmunology (2013) 2:e23794 10.4161/onci.23794 23734327 PMC3654597

[3] Balkwill F Mantovani A Inflammation and cancer: back to Virchow? Lancet (2001) 357:539–45 10.1016/S0140-6736(00)04046-0 11229684

[4] Hanahan D Weinberg RA Hallmarks of cancer: the next generation. Cell (2011) 144:646–74 10.1016/j.cell.2011.02.013 21376230

[5] Melero I Hervas-Stubbs S Glennie M Pardoll DM Chen L Immunostimulatory monoclonal antibodies for cancer therapy. Nat Rev Cancer (2007) 7:95–106 10.1038/nrc2051 17251916

[6] Hodi FS O’Day SJ McDermott DF Weber RW Sosman JA Haanen JB Improved survival with ipilimumab in patients with metastatic melanoma. N Engl J Med (2010) 363:711–23 10.1056/NEJMoa1003466 20525992 PMC3549297

[7] Topalian SL Hodi FS Brahmer JR Gettinger SN Smith DC McDermott DF Safety, activity, and immune correlates of anti-PD-1 antibody in cancer. N Engl J Med (2012) 366:2443–54 10.1056/NEJMoa1200690 22658127 PMC3544539

[8] Brahmer JR Tykodi SS Chow LQ Hwu WJ Topalian SL Hwu P Safety and activity of anti-PD-L1 antibody in patients with advanced cancer. N Engl J Med (2012) 366:2455–65 10.1056/NEJMoa1200694 22658128 PMC3563263

[9] Whiteside TL Inhibiting the inhibitors: evaluating agents targeting cancer immunosuppression. Expert Opin Biol Ther (2010) 10:1019–35 10.1517/14712598.2010.482207 20415597 PMC2882984

[10] Schroder K Hertzog PJ Ravasi T Hume DA Interferon-gamma: an overview of signals, mechanisms and functions. J Leukoc Biol (2004) 75:163–89 10.1189/jlb.0603252 14525967

[11] Ikeda H Old LJ Schreiber RD The roles of IFN gamma in protection against tumor development and cancer immunoediting. Cytokine Growth Factor Rev (2002) 13:95–109 10.1016/S1359-6101(01)00038-7 11900986

[12] Seliger B Ruiz-Cabello F Garrido F IFN inducibility of major histocompatibility antigens in tumors. Adv Cancer Res (2008) 101:249–76 10.1016/S0065-230X(08)00407-7 19055946 PMC7125809

[13] Kelchtermans H Billiau A Matthys P How interferon-gamma keeps autoimmune diseases in check. Trends Immunol (2008) 29:479–86 10.1016/j.it.2008.07.002 18775671

[14] Zhang J Yin and yang interplay of IFN-gamma in inflammation and autoimmune disease. J Clin Invest (2007) 117:871–3 10.1172/JCI31860 17404615 PMC1838954

[15] Stoicov C Fan X Liu JH Bowen G Whary M Kurt-Jones E T-bet knockout prevents *Helicobacter felis*-induced gastric cancer. J Immunol (2009) 183:642–9 10.4049/jimmunol.0900511 19535625 PMC3966469

[16] Syu LJ El-Zaatari M Eaton KA Liu Z Tetarbe M Keeley TM Transgenic expression of interferon-gamma in mouse stomach leads to inflammation, metaplasia, and dysplasia. Am J Pathol (2012) 181:2114–25 10.1016/j.ajpath.2012.08.017 23036899 PMC3509761

[17] Cho HI Lee YR Celis E Interferon gamma limits the effectiveness of melanoma peptide vaccines. Blood (2011) 117:135–44 10.1182/blood-2010-08-298117 20889921 PMC3037740

[18] Malmberg KJ Levitsky V Norell H de Matos CT Carlsten M Schedvins K IFN-gamma protects short-term ovarian carcinoma cell lines from CTL lysis via a CD94/NKG2A-dependent mechanism. J Clin Invest (2002) 110:1515–23 10.1172/JCI200215564 12438449 PMC151808

[19] Carswell EA Old LJ Kassel RL Green S Fiore N Williamson B An endotoxin-induced serum factor that causes necrosis of tumors. Proc Natl Acad Sci U S A (1975) 72:3666–70 10.1073/pnas.72.9.3666 1103152 PMC433057

[20] van Horssen R Ten Hagen TL Eggermont AM TNF-alpha in cancer treatment: molecular insights, antitumor effects, and clinical utility. Oncologist (2006) 11:397–408 10.1634/theoncologist.11-4-397 16614236

[21] Grivennikov SI Karin M Inflammatory cytokines in cancer: tumour necrosis factor and interleukin 6 take the stage. Ann Rheum Dis (2011) 70(Suppl 1):i104–8 10.1136/ard.2010.140145 21339211

[22] Gukovsky I Li N Todoric J Gukovskaya A Karin M Inflammation, autophagy, and obesity: common features in the pathogenesis of pancreatitis and pancreatic cancer. Gastroenterology (2013) 144:1199.e–209.e 10.1053/j.gastro.2013.02.007 23622129 PMC3786712

[23] Park EJ Lee JH Yu GY He G Ali SR Holzer RG Dietary and genetic obesity promote liver inflammation and tumorigenesis by enhancing IL-6 and TNF expression. Cell (2010) 140:197–208 10.1016/j.cell.2009.12.052 20141834 PMC2836922

[24] Balkwill F Tumour necrosis factor and cancer. Nat Rev Cancer (2009) 9:361–71 10.1038/nrc2628 19343034

[25] Wu Y Zhou BP TNF-alpha/NF-kappaB/Snail pathway in cancer cell migration and invasion. Br J Cancer (2010) 102:639–44 10.1038/sj.bjc.6605530 20087353 PMC2837572

[26] Achyut BR Bader DA Robles AI Wangsa D Harris CC Ried T Inflammation-mediated genetic and epigenetic alterations drive cancer development in the neighboring epithelium upon stromal abrogation of TGF-beta signaling. PLoS Genet (2013) 9:e1003251 10.1371/journal.pgen.1003251 23408900 PMC3567148

[27] Chaudhury A Howe PH The tale of transforming growth factor-beta (TGFbeta) signaling: a soigne enigma. IUBMB Life (2009) 61:929–39 10.1002/iub.239 19787707 PMC2810629

[28] Laouar Y Sutterwala FS Gorelik L Flavell RA Transforming growth factor-beta controls T helper type 1 cell development through regulation of natural killer cell interferon-gamma. Nat Immunol (2005) 6:600–7 10.1038/ni1197 15852008

[29] Prud’homme GJ Pathobiology of transforming growth factor beta in cancer, fibrosis and immunologic disease, and therapeutic considerations. Lab Invest (2007) 87:1077–91 10.1038/labinvest.3700669 17724448

[30] Ramalingam R Larmonier CB Thurston RD Midura-Kiela MT Zheng SG Ghishan FK Dendritic cell-specific disruption of TGF-beta receptor II leads to altered regulatory T cell phenotype and spontaneous multiorgan autoimmunity. J Immunol (2012) 189:3878–93 10.4049/jimmunol.1201029 22972928 PMC3466393

[31] Galdiero MR Garlanda C Jaillon S Marone G Mantovani A Tumor associated macrophages and neutrophils in tumor progression. J Cell Physiol (2013) 228:1404–12 10.1002/jcp.24260 23065796

[32] Moo-Young TA Larson JW Belt BA Tan MC Hawkins WG Eberlein TJ Tumor-derived TGF-beta mediates conversion of CD4+Foxp3+ regulatory T cells in a murine model of pancreas cancer. J Immunother (2009) 32:12–21 10.1097/CJI.0b013e318189f13c 19307989 PMC3862184

[33] Yang P Li QJ Feng Y Zhang Y Markowitz GJ Ning S TGF-beta-miR-34a-CCL22 signaling-induced Treg cell recruitment promotes venous metastases of HBV-positive hepatocellular carcinoma. Cancer Cell (2012) 22:291–303 10.1016/j.ccr.2012.07.023 22975373 PMC3443566

[34] Yang L TGFbeta and cancer metastasis: an inflammation link. Cancer Metastasis Rev (2010) 29:263–71 10.1007/s10555-010-9226-3 20437081 PMC6640647

[35] Massague J TGFbeta in cancer. Cell (2008) 134:215–30 10.1016/j.cell.2008.07.001 18662538 PMC3512574

[36] Kurebayashi Y Nagai S Ikejiri A Koyasu S Recent advances in understanding the molecular mechanisms of the development and function of Th17 cells. Genes Cells (2013) 18:247–65 10.1111/gtc.12039 23383714 PMC3657121

[37] Zou W Restifo NP T(H)17 cells in tumour immunity and immunotherapy. Nat Rev Immunol (2010) 10:248–56 10.1038/nri2742 20336152 PMC3242804

[38] Kaiga T Sato M Kaneda H Iwakura Y Takayama T Tahara H Systemic administration of IL-23 induces potent antitumor immunity primarily mediated through Th1-type response in association with the endogenously expressed IL-12. J Immunol (2007) 178:7571–80 17548592 10.4049/jimmunol.178.12.7571

[39] Kryczek I Wei S Szeliga W Vatan L Zou W Endogenous IL-17 contributes to reduced tumor growth and metastasis. Blood (2009) 114:357–9 10.1182/blood-2008-09-177360 19289853 PMC2714210

[40] Martin-Orozco N Muranski P Chung Y Yang XO Yamazaki T Lu S T helper 17 cells promote cytotoxic T cell activation in tumor immunity. Immunity (2009) 31:787–98 10.1016/j.immuni.2009.09.014 19879162 PMC2787786

[41] Nuñez S Saez JJ Fernandez D Flores-Santibanez F Alvarez K Tejon G T helper type 17 cells contribute to anti-tumour immunity and promote the recruitment of T helper type 1 cells to the tumour. Immunology (2013) 139:61–71 10.1111/imm.12055 23278668 PMC3634539

[42] Amedei A Niccolai E Benagiano M Della Bella C Cianchi F Bechi P Ex vivo analysis of pancreatic cancer-infiltrating T lymphocytes reveals that ENO-specific Tregs accumulate in tumor tissue and inhibit Th1/Th17 effector cell functions. Cancer Immunol Immunother (2013) 62:1249–60 10.1007/s00262-013-1429-3 23640603 PMC11028529

[43] Huang Z van Velkinburgh JC Ni B Wu Y Pivotal roles of the interleukin-23/T helper 17 cell axis in hepatitis B. Liver Int (2012) 32:894–901 10.1111/j.1478-3231.2012.02764.x 22340646

[44] Wu W Li J Chen F Zhu H Peng G Chen Z Circulating Th17 cells frequency is associated with the disease progression in HBV infected patients. J Gastroenterol Hepatol (2010) 25:750–7 10.1111/j.1440-1746.2009.06154.x 20492330

[45] Yang W Ding X Deng J Lu Y Matsuda Z Thiel A Interferon-gamma negatively regulates Th17-mediated immunopathology during mouse hepatitis virus infection. J Mol Med (Berl) (2011) 89:399–409 10.1007/s00109-010-0711-5 21191565 PMC7079994

[46] Ye Y Xie X Yu J Zhou L Xie H Jiang G Involvement of Th17 and Th1 effector responses in patients with Hepatitis B. J Clin Immunol (2010) 30:546–55 10.1007/s10875-010-9416-3 20393789

[47] He D Li H Yusuf N Elmets CA Li J Mountz JD IL-17 promotes tumor development through the induction of tumor promoting microenvironments at tumor sites and myeloid-derived suppressor cells. J Immunol (2010) 184:2281–8 10.4049/jimmunol.0902574 20118280 PMC3179912

[48] Qian X Gu L Ning H Zhang Y Hsueh EC Fu M Increased Th17 cells in the tumor microenvironment is mediated by IL-23 via tumor-secreted prostaglandin E2. J Immunol (2013) 190:5894–902 10.4049/jimmunol.1203141 23645882 PMC3660540

[49] Baird AM Leonard J Naicker KM Kilmartin L O’Byrne KJ Gray SG IL-23 is pro-proliferative, epigenetically regulated and modulated by chemotherapy in non-small cell lung cancer. Lung Cancer (2013) 79:83–90 10.1016/j.lungcan.2012.10.003 23116756

[50] Fukuda M Ehara M Suzuki S Ohmori Y Sakashita H IL-23 promotes growth and proliferation in human squamous cell carcinoma of the oral cavity. Int J Oncol (2010) 36:1355–65 10.3892/ijo_00000620 20428758

[51] Li J Zhang L Zhang J Wei Y Li K Huang L Interleukin 23 regulates proliferation of lung cancer cells in a concentration-dependent way in association with the interleukin-23 receptor. Carcinogenesis (2013) 34:658–66 10.1093/carcin/bgs384 23250909

[52] De Monte L Reni M Tassi E Clavenna D Papa I Recalde H Intratumor T helper type 2 cell infiltrate correlates with cancer-associated fibroblast thymic stromal lymphopoietin production and reduced survival in pancreatic cancer. J Exp Med (2011) 208:469–78 10.1084/jem.20101876 21339327 PMC3058573

[53] Aspord C Pedroza-Gonzalez A Gallegos M Tindle S Burton EC Su D Breast cancer instructs dendritic cells to prime interleukin 13-secreting CD4+ T cells that facilitate tumor development. J Exp Med (2007) 204:1037–47 10.1084/jem.20061120 17438063 PMC2118566

[54] Hallett MA Venmar KT Fingleton B Cytokine stimulation of epithelial cancer cells: the similar and divergent functions of IL-4 and IL-13. Cancer Res (2012) 72:6338–43 10.1158/0008-5472.CAN-12-3544 23222300 PMC3531868

[55] Koller FL Hwang DG Dozier EA Fingleton B Epithelial interleukin-4 receptor expression promotes colon tumor growth. Carcinogenesis (2010) 31:1010–7 10.1093/carcin/bgq044 20176658 PMC2878360

[56] Todaro M Alea MP Di Stefano AB Cammareri P Vermeulen L Iovino F Colon cancer stem cells dictate tumor growth and resist cell death by production of interleukin-4. Cell Stem Cell (2007) 1:389–402 10.1016/j.stem.2007.08.001 18371377

[57] Barderas R Bartolome RA Fernandez-Acenero MJ Torres S Casal JI High expression of IL-13 receptor alpha2 in colorectal cancer is associated with invasion, liver metastasis, and poor prognosis. Cancer Res (2012) 72:2780–90 10.1158/0008-5472.CAN-11-4090 22505647

[58] Fujisawa T Joshi B Nakajima A Puri RK A novel role of interleukin-13 receptor alpha2 in pancreatic cancer invasion and metastasis. Cancer Res (2009) 69:8678–85 10.1158/0008-5472.CAN-09-2100 19887609

[59] Fujisawa T Joshi BH Puri RK IL-13 regulates cancer invasion and metastasis through IL-13Ralpha2 via ERK/AP-1 pathway in mouse model of human ovarian cancer. Int J Cancer (2012) 131:344–56 10.1002/ijc.26366 21858811

[60] Virchow RLK Cellular Pathology as based upon Physiological and Pathological Histology. Philadelphia: J. B. Lippincott (1863). 10.1111/j.1753-4887.1989.tb02747.x 2649802

[61] Gabrilovich DI Nagaraj S Myeloid-derived suppressor cells as regulators of the immune system. Nat Rev Immunol (2009) 9:162–74 10.1038/nri2506 19197294 PMC2828349

[62] Qian BZ Pollard JW Macrophage diversity enhances tumor progression and metastasis. Cell (2010) 141:39–51 10.1016/j.cell.2010.03.014 20371344 PMC4994190

[63] Ammirante M Luo JL Grivennikov S Nedospasov S Karin M B-cell-derived lymphotoxin promotes castration-resistant prostate cancer. Nature (2010) 464:302–5 10.1038/nature08782 20220849 PMC2866639

[64] He Y Zha J Wang Y Liu W Yang X Yu P Tissue damage-associated “danger signals” influence T-cell responses that promote the progression of preneoplasia to cancer. Cancer Res (2013) 73:629–39 10.1158/0008-5472.CAN-12-2704 23108142

[65] Wang L Yi T Kortylewski M Pardoll DM Zeng D Yu H IL-17 can promote tumor growth through an IL-6-Stat3 signaling pathway. J Exp Med (2009) 206:1457–64 10.1084/jem.20090207 19564351 PMC2715087

[66] Devarajan P Chen Z Autoimmune effector memory T cells: the bad and the good. Immunol Res (2013) 57:12–22 10.1007/s12026-013-8448-1 24203440 PMC4067599

[67] Germain RN Robey EA Cahalan MD A decade of imaging cellular motility and interaction dynamics in the immune system. Science (2012) 336:1676–81 10.1126/science.1221063 22745423 PMC3405774

[68] Miska J Abdulreda MH Devarajan P Lui JB Suzuki J Pileggi A Real-time immune cell interactions in target tissue during autoimmune-induced damage and graft tolerance. J Exp Med (2014) 211:441–56 10.1084/jem.20130785 24567447 PMC3949570

[69] Suzuki J Ricordi C Chen Z Immune tolerance induction by integrating innate and adaptive immune regulators. Cell Transplant (2010) 19:253–68 10.3727/096368909X480314 19919733 PMC2884065

[70] Tomsova M Melichar B Sedlakova I Steiner I Prognostic significance of CD3+ tumor-infiltrating lymphocytes in ovarian carcinoma. Gynecol Oncol (2008) 108:415–20 10.1016/j.ygyno.2007.10.016 18037158

[71] Tano T Okamoto M Kan S Nakashiro K Shimodaira S Koido S Prognostic impact of expression of Bcl-2 and Bax genes in circulating immune cells derived from patients with head and neck carcinoma. Neoplasia (2013) 15:305–14 10.1593/neo.121528 23479508 PMC3593153

[72] Lippitz BE Cytokine patterns in patients with cancer: a systematic review. Lancet Oncol (2013) 14:e218–28 10.1016/S1470-2045(12)70582-X 23639322

[73] Vierboom MP Nijman HW Offringa R van der Voort EI van Hall T van den Broek L Tumor eradication by wild-type p53-specific cytotoxic T lymphocytes. J Exp Med (1997) 186:695–704 10.1084/jem.186.5.695 9271585 PMC2199025

[74] Hilburger Ryan M Abrams SI Characterization of CD8+ cytotoxic T lymphocyte/tumor cell interactions reflecting recognition of an endogenously expressed murine wild-type p53 determinant. Cancer Immunol Immunother (2001) 49:603–12 10.1007/s002620000156 11225991 PMC11036958

[75] Hu J Kindsvogel W Busby S Bailey MC Shi YY Greenberg PD An evaluation of the potential to use tumor-associated antigens as targets for antitumor T cell therapy using transgenic mice expressing a retroviral tumor antigen in normal lymphoid tissues. J Exp Med (1993) 177:1681–90 10.1084/jem.177.6.1681 8496686 PMC2191055

[76] Morgan DJ Kreuwel HT Fleck S Levitsky HI Pardoll DM Sherman LA Activation of low avidity CTL specific for a self epitope results in tumor rejection but not autoimmunity. J Immunol (1998) 160:643–51 9551898

[77] Caspi RR Immunotherapy of autoimmunity and cancer: the penalty for success. Nat Rev Immunol (2008) 8:970–6 10.1038/nri2438 19008897 PMC2764117

[78] Attia P Phan GQ Maker AV Robinson MR Quezado MM Yang JC Autoimmunity correlates with tumor regression in patients with metastatic melanoma treated with anti-cytotoxic T-lymphocyte antigen-4. J Clin Oncol (2005) 23:6043–53 10.1200/JCO.2005.06.205 16087944 PMC1473965

[79] Andersen MH Junker N Ellebaek E Svane IM Thor Straten P Therapeutic cancer vaccines in combination with conventional therapy. J Biomed Biotechnol (2010) 2010:237623 10.1155/2010/237623 20617155 PMC2896846

[80] Zaidi MR Merlino G The two faces of interferon-gamma in cancer. Clin Cancer Res (2011) 17:6118–24 10.1158/1078-0432.CCR-11-0482 21705455 PMC3186825

[81] Fojtova M Boudny V Kovarik A Lauerova L Adamkova L Souckova K Development of IFN-gamma resistance is associated with attenuation of SOCS genes induction and constitutive expression of SOCS 3 in melanoma cells. Br J Cancer (2007) 97:231–7 10.1038/sj.bjc.6603849 17579625 PMC2360293

[82] Miller CH Maher SG Young HA Clinical use of interferon-gamma. Ann N Y Acad Sci (2009) 1182:69–79 10.1111/j.1749-6632.2009.05069.x 20074276 PMC6574079

[83] Demaria S Pikarsky E Karin M Coussens LM Chen YC El-Omar EM Cancer and inflammation: promise for biologic therapy. J Immunother (2010) 33:335–51 10.1097/CJI.0b013e3181d32e74 20386472 PMC2941912

[84] Moss SF Blaser MJ Mechanisms of disease: Inflammation and the origins of cancer. Nat Clin Pract Oncol (2005) 2:90–7 quiz 91 p following 113, 10.1038/ncponc0081 16264881

[85] Skurkovich B Skurkovich S Anti-interferon-gamma antibodies in the treatment of autoimmune diseases. Curr Opin Mol Ther (2003) 5:52–7 12669471

[86] Skurkovich S Skurkovich B Kelly J Anticytokine therapy, particularly anti-IFN-gamma, in Th1-mediated autoimmune diseases. Expert Rev Clin Immunol (2005) 1:11–25 10.1586/1744666X.1.1.11 20477651

[87] Fox JG Wang TC Inflammation, atrophy, and gastric cancer. J Clin Invest (2007) 117:60–9 10.1172/JCI30111 17200707 PMC1716216

[88] Merchant JL What lurks beneath: IL-11, via Stat3, promotes inflammation-associated gastric tumorigenesis. J Clin Invest (2008) 118:1628–31 10.1172/JCI35344 18431518 PMC2323194

[89] Polk DB Peek RM Jr *Helicobacter pylori*: gastric cancer and beyond. Nat Rev Cancer (2010) 10:403–14 10.1038/nrc2857 20495574 PMC2957472

[90] Toh BH van Driel IR Gleeson PA Pernicious anemia. N Engl J Med (1997) 337:1441–8 10.1056/NEJM199711133372007 9358143

[91] El-Omar EM Rabkin CS Gammon MD Vaughan TL Risch HA Schoenberg JB Increased risk of noncardia gastric cancer associated with proinflammatory cytokine gene polymorphisms. Gastroenterology (2003) 124:1193–201 10.1016/S0016-5085(03)00157-4 12730860

[92] Judd LM Alderman BM Howlett M Shulkes A Dow C Moverley J Gastric cancer development in mice lacking the SHP2 binding site on the IL-6 family co-receptor gp130. Gastroenterology (2004) 126:196–207 10.1053/j.gastro.2003.10.066 14699500

[93] Tu S Bhagat G Cui G Takaishi S Kurt-Jones EA Rickman B Overexpression of interleukin-1beta induces gastric inflammation and cancer and mobilizes myeloid-derived suppressor cells in mice. Cancer Cell (2008) 14:408–19 10.1016/j.ccr.2008.10.011 18977329 PMC2586894

[94] Weis VG Goldenring JR Current understanding of SPEM and its standing in the preneoplastic process. Gastric Cancer (2009) 12:189–97 10.1007/s10120-009-0527-6 20047123 PMC4502916

[95] Szabo SJ Kim ST Costa GL Zhang X Fathman CG Glimcher LH A novel transcription factor, T-bet, directs Th1 lineage commitment. Cell (2000) 100:655–69 10.1016/S0092-8674(00)80702-3 10761931

[96] Li X McKinstry KK Swain SL Dalton DK IFN-gamma acts directly on activated CD4+ T cells during mycobacterial infection to promote apoptosis by inducing components of the intracellular apoptosis machinery and by inducing extracellular proapoptotic signals. J Immunol (2007) 179:939–49 17617585 10.4049/jimmunol.179.2.939 PMC2532516

[97] Mellor AL Munn DH Creating immune privilege: active local suppression that benefits friends, but protects foes. Nat Rev Immunol (2008) 8:74–80 10.1038/nri2233 18064049

[98] Tu SP Quante M Bhagat G Takaishi S Cui G Yang XD IFN-gamma inhibits gastric carcinogenesis by inducing epithelial cell autophagy and T-cell apoptosis. Cancer Res (2011) 71:4247–59 10.1158/0008-5472.CAN-10-4009 21512143 PMC3139967

[99] White E DiPaola RS The double-edged sword of autophagy modulation in cancer. Clin Cancer Res (2009) 15:5308–16 10.1158/1078-0432.CCR-07-5023 19706824 PMC2737083

[100] Bradley JR TNF-mediated inflammatory disease. J Pathol (2008) 214:149–60 10.1002/path.2287 18161752

[101] Palladino MA Bahjat FR Theodorakis EA Moldawer LL Anti-TNF-alpha therapies: the next generation. Nat Rev Drug Discov (2003) 2:736–46 10.1038/nrd1175 12951580

[102] Murdaca G Colombo BM Cagnati P Gulli R Spano F Puppo F Update upon efficacy and safety of TNF-alpha inhibitors. Expert Opin Drug Saf (2012) 11:1–5 10.1517/14740338.2012.630388 22010813

[103] Hobohm U Fever and cancer in perspective. Cancer Immunol Immunother (2001) 50:391–6 11726133 10.1007/s002620100216 PMC11032960

[104] Kruglov AA Kuchmiy A Grivennikov SI Tumanov AV Kuprash DV Nedospasov SA Physiological functions of tumor necrosis factor and the consequences of its pathologic overexpression or blockade: mouse models. Cytokine Growth Factor Rev (2008) 19:231–44 10.1016/j.cytogfr.2008.04.010 18502680

[105] Moore RJ Owens DM Stamp G Arnott C Burke F East N Mice deficient in tumor necrosis factor-alpha are resistant to skin carcinogenesis. Nat Med (1999) 5:828–31 10.1038/10552 10395330

[106] Fujiki H Sueoka E Suganuma M Tumor promoters: from chemicals to inflammatory proteins. J Cancer Res Clin Oncol (2013) 139:1603–14 10.1007/s00432-013-1455-8 23756937 PMC11824212

[107] Capece D Fischietti M Verzella D Gaggiano A Cicciarelli G Tessitore A The inflammatory microenvironment in hepatocellular carcinoma: a pivotal role for tumor-associated macrophages. Biomed Res Int (2013) 2013:187204 10.1155/2013/187204 23533994 PMC3591180

[108] Natarajan M Gibbons CF Mohan S Moore S Kadhim MA Oxidative stress signalling: a potential mediator of tumour necrosis factor alpha-induced genomic instability in primary vascular endothelial cells. Br J Radiol (2007) 80(1):S13–22 10.1259/bjr/15316848 17704321

[109] Pierce RH Campbell JS Stephenson AB Franklin CC Chaisson M Poot M Disruption of redox homeostasis in tumor necrosis factor-induced apoptosis in a murine hepatocyte cell line. Am J Pathol (2000) 157:221–36 10.1016/S0002-9440(10)64533-6 10880392 PMC1850198

[110] Suematsu N Tsutsui H Wen J Kang D Ikeuchi M Ide T Oxidative stress mediates tumor necrosis factor-alpha-induced mitochondrial DNA damage and dysfunction in cardiac myocytes. Circulation (2003) 107:1418–23 10.1161/01.CIR.0000055318.09997.1F 12642364

[111] Wheelhouse NM Chan YS Gillies SE Caldwell H Ross JA Harrison DJ TNF-alpha induced DNA damage in primary murine hepatocytes. Int J Mol Med (2003) 12:889–94 10.3892/ijmm.12.6.889 14612962

[112] Li MO Flavell RA TGF-beta: a master of all T cell trades. Cell (2008) 134:392–404 10.1016/j.cell.2008.07.025 18692464 PMC3677783

[113] Kulkarni AB Huh CG Becker D Geiser A Lyght M Flanders KC Transforming growth factor beta 1 null mutation in mice causes excessive inflammatory response and early death. Proc Natl Acad Sci U S A (1993) 90:770–4 10.1073/pnas.90.2.770 8421714 PMC45747

[114] Leveen P Larsson J Ehinger M Cilio CM Sundler M Sjostrand LJ Induced disruption of the transforming growth factor beta type II receptor gene in mice causes a lethal inflammatory disorder that is transplantable. Blood (2002) 100:560–8 10.1182/blood.V100.2.560 12091349

[115] Ni XY Sui HX Liu Y Ke SZ Wang YN Gao FG TGF-beta of lung cancer microenvironment upregulates B7H1 and GITRL expression in dendritic cells and is associated with regulatory T cell generation. Oncol Rep (2012) 28:615–21 10.3892/or.2012.1822 22614805

[116] Wilson CA Cajulis EE Green JL Olsen TM Chung YA Damore MA HER-2 overexpression differentially alters transforming growth factor-beta responses in luminal versus mesenchymal human breast cancer cells. Breast Cancer Res (2005) 7:R1058–79 10.1186/bcr1343 16457687 PMC1410754

[117] Baldwin RL Tran H Karlan BY Loss of c-myc repression coincides with ovarian cancer resistance to transforming growth factor beta growth arrest independent of transforming growth factor beta/Smad signaling. Cancer Res (2003) 63:1413–9 12649207

[118] Connolly EC Freimuth J Akhurst RJ Complexities of TGF-beta targeted cancer therapy. Int J Biol Sci (2012) 8:964–78 10.7150/ijbs.4564 22811618 PMC3399319

[119] Connolly EC Saunier EF Quigley D Luu MT De Sapio A Hann B Outgrowth of drug-resistant carcinomas expressing markers of tumor aggression after long-term TbetaRI/II kinase inhibition with LY2109761. Cancer Res (2011) 71:2339–49 10.1158/0008-5472.CAN-10-2941 21282335 PMC3059399

[120] Ishigame H Kakuta S Nagai T Kadoki M Nambu A Komiyama Y Differential roles of interleukin-17A and -17F in host defense against mucoepithelial bacterial infection and allergic responses. Immunity (2009) 30:108–19 10.1016/j.immuni.2008.11.009 19144317

[121] Ye J Livergood RS Peng G The role and regulation of human Th17 cells in tumor immunity. Am J Pathol (2013) 182:10–20 10.1016/j.ajpath.2012.08.041 23159950 PMC3532708

[122] Yosef N Shalek AK Gaublomme JT Jin H Lee Y Awasthi A Dynamic regulatory network controlling TH17 cell differentiation. Nature (2013) 496:461–8 10.1038/nature11981 23467089 PMC3637864

[123] Harrington LE Hatton RD Mangan PR Turner H Murphy TL Murphy KM Interleukin 17-producing CD4+ effector T cells develop via a lineage distinct from the T helper type 1 and 2 lineages. Nat Immunol (2005) 6:1123–32 10.1038/ni1254 16200070

[124] Park H Li Z Yang XO Chang SH Nurieva R Wang YH A distinct lineage of CD4 T cells regulates tissue inflammation by producing interleukin 17. Nat Immunol (2005) 6:1133–41 10.1038/ni1261 16200068 PMC1618871

[125] Langowski JL Kastelein RA Oft M Swords into plowshares: IL-23 repurposes tumor immune surveillance. Trends Immunol (2007) 28:207–12 10.1016/j.it.2007.03.006 17395538

[126] McKenzie BS Kastelein RA Cua DJ Understanding the IL-23-IL-17 immune pathway. Trends Immunol (2006) 27:17–23 10.1016/j.it.2005.10.003 16290228

[127] Murugaiyan G Saha B Protumor vs antitumor functions of IL-17. J Immunol (2009) 183:4169–75 10.4049/jimmunol.0901017 19767566

[128] Oppmann B Lesley R Blom B Timans JC Xu Y Hunte B Novel p19 protein engages IL-12p40 to form a cytokine, IL-23, with biological activities similar as well as distinct from IL-12. Immunity (2000) 13:715–25 10.1016/S1074-7613(00)00070-4 11114383

[129] Hatton RD TGF-beta in Th17 cell development: the truth is out there. Immunity (2011) 34:288–90 10.1016/j.immuni.2011.03.009 21435582 PMC3097895

[130] Langowski JL Zhang X Wu L Mattson JD Chen T Smith K IL-23 promotes tumour incidence and growth. Nature (2006) 442:461–5 10.1038/nature04808 16688182

[131] Toussirot E The IL23/Th17 pathway as a therapeutic target in chronic inflammatory diseases. Inflamm Allergy Drug Targets (2012) 11:159–68 10.2174/187152812800392805 22280236

[132] Van Dyken SJ Locksley RM Interleukin-4- and interleukin-13-mediated alternatively activated macrophages: roles in homeostasis and disease. Annu Rev Immunol (2013) 31:317–43 10.1146/annurev-immunol-032712-095906 23298208 PMC3606684

[133] Chapoval S Dasgupta P Dorsey NJ Keegan AD Regulation of the T helper cell type 2 (Th2)/T regulatory cell (Treg) balance by IL-4 and STAT6. J Leukoc Biol (2010) 87:1011–8 10.1189/jlb.1209772 20335310 PMC2872539

[134] Quatromoni JG Eruslanov E Tumor-associated macrophages: function, phenotype, and link to prognosis in human lung cancer. Am J Transl Res (2012) 4:376–89 23145206 PMC3493031

[135] LaPorte SL Juo ZS Vaclavikova J Colf LA Qi X Heller NM Molecular and structural basis of cytokine receptor pleiotropy in the interleukin-4/13 system. Cell (2008) 132:259–72 10.1016/j.cell.2007.12.030 18243101 PMC2265076

[136] Oh CK Geba GP Molfino N Investigational therapeutics targeting the IL-4/IL-13/STAT-6 pathway for the treatment of asthma. Eur Respir Rev (2010) 19:46–54 10.1183/09059180.00007609 20956165 PMC9491642

[137] Li Z Jiang J Wang Z Zhang J Xiao M Wang C Endogenous interleukin-4 promotes tumor development by increasing tumor cell resistance to apoptosis. Cancer Res (2008) 68:8687–94 10.1158/0008-5472.CAN-08-0449 18974110

[138] Humphries A Cereser B Gay LJ Miller DS Das B Gutteridge A Lineage tracing reveals multipotent stem cells maintain human adenomas and the pattern of clonal expansion in tumor evolution. Proc Natl Acad Sci U S A (2013) 110:E2490–9 10.1073/pnas.1220353110 23766371 PMC3704042

[139] Schepers AG Snippert HJ Stange DE van den Born M van Es JH van de Wetering M Lineage tracing reveals Lgr5+ stem cell activity in mouse intestinal adenomas. Science (2012) 337:730–5 10.1126/science.1224676 22855427

[140] Todaro M Perez Alea M Scopelliti A Medema JP Stassi G IL-4-mediated drug resistance in colon cancer stem cells. Cell Cycle (2008) 7:309–13 10.4161/cc.7.3.5389 18235245

[141] Hershey GK IL-13 receptors and signaling pathways: an evolving web. J Allergy Clin Immunol (2003) 111:677–90 quiz 691, 10.1067/mai.2003.1333 12704343

[142] Shimamura T Fujisawa T Husain SR Joshi B Puri RK Interleukin 13 mediates signal transduction through interleukin 13 receptor alpha2 in pancreatic ductal adenocarcinoma: role of IL-13 pseudomonas exotoxin in pancreatic cancer therapy. Clin Cancer Res (2010) 16:577–86 10.1158/1078-0432.CCR-09-2015 20068108

[143] Hall B Nakashima H Sun ZJ Sato Y Bian Y Husain SR Targeting of interleukin-13 receptor alpha2 for treatment of head and neck squamous cell carcinoma induced by conditional deletion of TGF-beta and PTEN signaling. J Transl Med (2013) 11:45 10.1186/1479-5876-11-45 23421960 PMC3598213

[144] Shimamura T Husain SR Puri RK The IL-4 and IL-13 pseudomonas exotoxins: new hope for brain tumor therapy. Neurosurg Focus (2006) 20:E11 10.3171/foc.2006.20.4.6 16709016

[145] Mut M Sherman JH Shaffrey ME Schiff D Cintredekin besudotox in treatment of malignant glioma. Expert Opin Biol Ther (2008) 8:805–12 10.1517/14712598.8.6.805 18476792

[146] Ohka F Natsume A Wakabayashi T Current trends in targeted therapies for glioblastoma multiforme. Neurol Res Int (2012) 2012:878425 10.1155/2012/878425 22530127 PMC3317017

[147] Han D Cai X Wen J Kenyon NS Chen Z From biomarkers to a clue of biology: a computation-aided perspective of immune gene expression profiles in human type 1 diabetes. Front Immunol (2012) 3:320 10.3389/fimmu.2012.00320 23112798 PMC3480653

[148] Liseth K Ersvaer E Hervig T Bruserud O Combination of intensive chemotherapy and anticancer vaccines in the treatment of human malignancies: the hematological experience. J Biomed Biotechnol (2010) 2010:692097 10.1155/2010/692097 20625438 PMC2896720

[149] Finn OJ Cancer immunology. N Engl J Med (2008) 358:2704–15 10.1056/NEJMra072739 18565863

[150] Braumüller H Wieder T Brenner E Assmann S Hahn M Alkhaled M T-helper-1-cell cytokines drive cancer into senescence. Nature (2013) 494:361–5 10.1038/nature11824 23376950

